# Can a Well-Being Economy Save Us?

**DOI:** 10.34172/ijhpm.8507

**Published:** 2024-06-19

**Authors:** Ronald Labonté

**Affiliations:** School of Epidemiology and Public Health, Faculty of Medicine, University of Ottawa, Ottawa, ON, Canada.

**Keywords:** Political Economy, Well-Being Economics, Post-growth Economics

## Abstract

The COVID-19 pandemic led many countries to consider reforms to their economic policies, in part to better deal with global warming, mass population migration and displacements, and worsening global inequalities. Some health progressive changes have been made, but the world still confronts the contradiction between economic growth and the need to reduce aggregate global consumption. Well-being economies based on valuing human and planetary health have been proposed as a viable option, with more appeal than concepts such as degrowth or postgrowth economics. Some governments are moving in a "well-being economy" direction, but are they moving far and fast enough? What are the policy actions governments must take, and how will they overcome powerful interests opposed to any economic changes that might challenge their privileges? The idea of well-being economies resonates strongly with most cultures; and therein lies its civil society activist potential.

 The COVID-19 pandemic once again brought attention to the systemic failures of our global economies to sustain human and environmental “health for all.” Elsewhere I commented on the “build back better” policies (notably those of the United States and the European Union) noting some gains but also systemic limitations largely owing to the power of corporate lobbies, polarized politics, and increasingly autocratic regimes on both sides of the Atlantic.^1^ The Russian invasion of Ukraine and the Israel/Gaza war have not made the geopolitics of incremental reform any easier.

 I also wrote of the rise of degrowth and postgrowth economics that argued the need to pivot away from our disequalizing capitalism (market, state, or otherwise) and its toxic obsession with growth and consumption and towards an economics premised on promoting human and planetary well-being.^2^ In this editorial I discuss the forces for and against such an outcome.

## What Is a Well-Being Economy?

 In simplest terms, a well-being economy is one that pursues an equitable global allocation of the resources people need for a healthy life while staying within the ecological limits of our planet.^3^ Some extend this to minimizing the impacts of human activities on all other living species, rewilding our natural surroundings, and upending the current human-generated sixth mass extinction.^4^ While sharing some commonalities with earlier-generation welfare economics and its goal of maximizing people’s overall social satisfaction through cost-benefit analysis and social welfare functions,^5^ well-being economics differs in several important ways, eg, environmental concerns are more central, reducing wealth and power inequalities replaces economic efficiency, and conventional notions of economic growth are interrogated for their coherence with justice and human rights norms. Well-being economics also draws on the economic concept of “public goods” (such as air, water, biodiversity, and peace) access to which are considered non-excludable (everyone can access them) and non-rivalrous (use by one person does not prevent use by another). Unlike market-based private goods, public goods, often referred to as common goods, require regulatory and redistributive or pre-distributive measures by governments to offset or prevent market failures.^6^

 The emphasis on protecting the environmental commons in where well-being economics and welfare economics most clearly part company. Since the 1987 United Nations Brundtland Commission’s call for a “sustainable development” that met “the needs of the present without compromising the ability of future generations to meet their own needs,” the tension between environmental protection and market-driven economic growth (however welfare optimized) has persisted in national and global policy discourse. It is helpful to note the emphasis the Brundtland Report’s definition of sustainable development placed on “needs” (sufficiency) and not “wants” (the ad-incentivized and status-driven excess consumption that characterizes the world’s wealthier nations and is now rapidly globalizing). This reflects the empirical reality that continuous increases in consumption on a planet of finite resources is not possible, but an equitable distribution of resources essential for life is.

## The World Health Organization Rediscovers Well-Being

 Brundtland, a few years after the Commission that bears her name, became World Health Organization (WHO) Director-General. One of her legacies is the 2001 Commission on Macroeconomics and Health^7^ which made the investment case for public health as a necessary engine for economic growth. Missing then (as in much liberal or neoliberal economics today) was any consideration of the negative environmental externalities of such growth, and an ahistorical and acritical acceptance of market fundamentalism.^8^ The 2008 report of the WHO Commission on Social Determinants of Health^9^ was more critical in its claim that health inequalities resulted from “a toxic combination of poor social policies and programs, unfair economic arrangements, and bad politics,” but had yet to encounter the deeper economic critiques of the Anthropocene’s ecological devastations. There was little WHO chatter of “well-being” apart from the obligatory nod to its founding statement of health being “a state of complete physical, mental and social well-being” (to which “spiritual” is sometimes appended) until a few years ago.

 The WHO Council on the Economics of Health For All (2021-2023) was established by current Director-General Tedros to define the basic principles of a political economy centered on creating equitable “health for all.” Its final report encourages governments to direct their economies towards social and planetary well-being rather than economic growth, beginning with adopting or adapting new measures beyond gross domestic product (GDP).^10^ The Council’s 13 high-level recommendations, if implemented, would “transform economic systems and co-create an economic policy design guide to shift societal success beyond GDP growth and instead deliver shared well-being.”^11^ At minimum this calls for an overhaul of national and international systems for health financing in which health is no longer seen simply as an investment to grow the economy, but the economy is purposefully reshaped to achieve democratically decided upon health, social, and environmental goals. Economic growth *per se* would be subordinated to achieving these goals, rather than the other way around.

 In parallel to the Council’s work the WHO in 2022 developed a framework for creation of a well-being economy, one which, echoing degrowth and postgrowth arguments discussed in one of my earlier articles,^12^ would move away from economic models “based on massive and intensive production and consumption of goods.”^13^ The normative policy advice it offers to WHO member states is a compendium of generally good ideas that range from blue-sky economic ideals to public health-policy specifics, although unlike the Council’s work it is silent on how such ideas might be financed or implemented. As such it comprises a commendable wish list of policy suggestions ranging from the more (potentially) transformative to the rather mundane.

## Well-Being for an Earth for All

 Around the same time as these two WHO reports were advancing a well-being agenda, the *Earth for All *(E4A) collaboration released its “survival guide for humanity.”^14^ Updating the Club of Rome’s 1972 seminal report, *The Limits to Growth*,^15^ E4A identifies “five great turnarounds” needed for humanity to live in a fair and ecologically sustained space: ending poverty, addressing inequality, accelerating gender inequity, transforming food systems, and transitioning to green energy. Beneath these high-level aspirations are a number of specific policy measures for their achievement, many of them reflecting those long advocated by progressive social movements. The report models change in a well-being index and measures of poverty, inequality, social tension and observed warming to the year 2100, under two scenarios: a continuation of current trends (“too little, too late”) and rapid adoption of the policy levers for the five great turnarounds (“the great leap”). Only the second offers a livable future.

 Like the two WHO initiatives, the E4A report calls for governments to reshape markets to deliver a “well-being economy based on the commons” in which resources are managed collectively and democratically and new logics of production are created. Similar to points other critics of consumptive capitalism have made, the E4A report emphasizes the local scale at which such transformations are already taking place, such as “*seed-sharing cooperatives, communities of open-sourced software programmers, creation and use of complementary currencies to stimulate local economies…community-supported agriculture, rewilding…and community land trusts*” (p. 161).^14^ The survival challenge becomes scaling up and globally diffusing such initiatives (See Table for the headline well-being economy policy recommendations from all three reports).

**Table:** Author’s Summary of Complementary Policy Reforms Recent Well-Being Reports

**WHO Economics Council**	**WHO Well-Being Framework**	**Earth For All**
*Valuing Health for All*	*Nurture Planet Earth*	*Ending Poverty*
Treat health and well-being, health workers and health systems as investment, not cost	National action plans to achieve environmental goals (air, water, biodiversity, climate change)	Invest $1 trillion annually in green economy in LICs
Use legal and financial commitments to enforce health as a human right	Reduce fossil fuel extraction and relance	Cancel all debt in LICs (countries where per capita is less then $10 000 annually)
Uphold international commitments to a regenerative economy which links planet and people	Increase renewable alternatives and reduce energy consumption	Protect fledging industries in LICs, and reform intellectual proper rights to create technology transfer
Use metric beyond GDP to track progress on core societal values	Adopt commodity pricing to “make eco-friendly choices” more accessible	*Addressing Gross Inequality*
*Financing Health for All*	Adopt a One Health approach to sustainable food systems	Increase progressive corporate and personal taxation everywhere, and eliminate loopholes/tax havens
Suspend debt repayments by LICs, increase progressive taxation everywhere including wealth taxes	*Design Equitable and Inclusive Social Protection*	Legislate and enforce strong labour rights, extended to informal employment (“gig” work)
Reform global financing mechanisms to ensure >$1 trillion in transfers to LICs	Expand social welfare systems with adequate financing	Create “Citizens Funds” (eg, Universal Basic Income, Citizens’ Dividends) as tax-funded redistribution
Provide adequate funding for WHO to play its key role in coordinating Health for All policy transformations	Support transition for informal to formal economy (eg, strengthen labour rights)	*Creating Gender Equity*
*Innovating for Health*	Ensure social protection measures conform with environmental protection measures	Ensure education access for all girls/women worldwide
Build public-private partnerships with equity conditions on private partners (eg, where there is publicly financed R&D)	Improve gender equality and reduce stigma/discrimination and interpersonal violence	Ensure gender equity in jobs and political/economic leadership
Reform intellectual property regimes to ensure equitable access to new health discoveries and technologies	*Design and Support Equitable Economies*	Ensure adequate pensions for all
Orient government industrial strategies to deliver Health for All	Consider monetary and fiscal policies that reduce inequalities and prioritize health and planetary well-being	*Transforming Food Systems*
*Strengthening Public Sector*	Re-orient investments to place health at the centre of our value system	Legislate to reduce food loss/waste
Greater whole-of-government collaboration to achieve Health for All, emphasis on role of finance and economic ministries	Dis-incentive production and consumption of produces and services that harm population health	Implement regenerative agriculture (agroecology)
Invest in the innovative capacities of the public sector	Create fiscal space for investments in well-being, implement measures beyond GDP	Promote healthy diets within planetary boundaries (less meat, more vegetables)
Strengthen and expand the space for public engagement in political policy-making	Leverage the role of central and investment banks towards investing in well-being economies	*Transforming Energy Systems*
	*Promote Equitable Universal Health Coverage*	Immediate phase out of all fossil fuel subsidies, invest >$1 trillion annually in renewables*
	Reinforce primary health care approaches to health systems	Electrify everything
	Establish social infrastructures for public engagement in policy-making	Improve energy efficiency and storage at scale
	*Promote Equitable Digital Systems*	
	Control health-related misinformation/disinformation	
	Promote digital and media literacy, enhance protection of personal data	

Abbreviations: WHO, World Health Organization; LICs, low-income countries; GDP, gross domestic product; R&D, research and development. * The $1 trillion for energy transition away from fossil fuels is a low-ball estimate and in 2022 was already met. The International Renewable Energy Agency cautions that such investment must now be >$5 trillion annually if the 1.5C limit is to be kept. https://www.irena.org/Publications/2023/Mar/World-Energy-Transitions-Outlook-2023.

## Well-Being Economies in Practice

 Without government support, these forays into well-being economic thought leadership will ossify into nice ideas from the past or fade away in the *realpolitik *of global turmoil. But some efforts are underway. Since 2018 a small group of the world’s governments have been attempting to integrate human and ecological well-being into their fiscal (tax, regulatory, and spending) policies. In 2020, five countries formed the network of Wellbeing Economy Governments (WEGo): Finland, Scotland, Wales, Iceland, and New Zealand^16^; Canada is a sixth unofficial fellow traveller, and all are part of a Wellbeing Economy Alliance (WEALL), founded in 2018 and supported by hundreds of organizations, researchers, and local hubs, with the time-limited intent of catalyzing government and non-governmental actions that embody the tenets of human and ecological well-being.

 Since 2021 the Euro WHO office has supported development of policy-level understanding of well-being economies across the EU region, alongside other WEGo countries. There is momentum beyond governments. A WEALL (https://weall.org/), also established in 2018, has over 200 member organizations and thousands of individuals. Like UK economist Kate Raworth’s “doughnut economics action lab” (https://doughnuteconomics.org/) and its network of over 50 self-organizing local groups mapping socio-environmental performance, WEALL has several local hubs advancing the principles and practices of well-being economies. Unsurprisingly, Raworth’s doughnut economic model and well-being frameworks share much in common, as they do with other postgrowth and degrowth principles.

## But Is There Political Traction?

 Although the WEGo is the first time a group of national governments had given serious consideration to postgrowth economic strategies, evidence of substantial change in government budgeting or decision-making remains sparse. Most WEGo countries so far have been focusing primarily on developing and integrating alternative “beyond GDP” measures into their public accounts. This is not a new undertaking, with relatively recent efforts dating back to the social indicators movement of the 1980s and 1990s,^17^ culminating in the 1990 development (and ongoing refinement) of the United Nations Development Program’s Human Development Index. Nor are the WEGo countries and UN agencies alone in such efforts. By one account, a majority of member states of the Organization for Economic Co-operation and Development (the club of rich nations)^18^ are engaged in some developing or using some form of “well-being” assessment measures.

 Canada’s engagement with the well-being idea was mandating a quality-of-life impact assessment of its 2021 federal budget. Substantive application of the assessment, however, is still absent.^19^ Similar concerns exist with other countries, such as Australia’s lauded 2023 *Measuring What Matters *Framework.^20^ Other WEGo countries manifest their approach to well-being by emphasizing gender equality or experimenting with universal basic income programs. Earlier in 2015 (the year of the Sustainable Development Goals and the Paris Accord) Wales innovatively appointed a Future Generations Commissioner with a mandate to oversee the extent to which public bodies were safeguarding future generations’ needs. New Zealand, however, took the biggest leap forward. In 2019 it created the world’s first “well-being budget,” committing NZ $26 billion over four years to improve child health, create new employment, support mental health, provide opportunities for marginalized groups, and transition to a low-emission economy. Although this well-being allocation comprised just 5% of overall government spending, it was considered a small move in the right direction. With a change to a pro-business anti-tax conservative government in late 2023, it is moot whether the idea of a well-being budget will survive,^21^ much less its level of funding.

## The Risk of Performative Change Only

 Performativity, the theory that language can function as a form of social action and change, has been foundational to much social science study (and argumentation) since the 1950s. That language takes shape in, and shapes, actions in the world is not seriously challenged; but performativity (the uptake of language or concepts) can often lead to easy and costless actions that do not challenge the status quo. With respect to well-being economies the risk is that it devolves to a matter of creating and reporting on new well-being measures that may (or may not) have any significant impact on government decision-making or economic practices. It becomes performance rather than transformation.

 Even as performance there are limitations in the current well-being economies’ focus on measurement and frameworks, with each country or region engaged in creating its own template. While a global consensus on measurement is likely neither possible nor desirable (along the lines of the accepted global environmental norm of “common but differentiated responsibilities” reflecting different national priorities or capacities) a plethora of differing metrics and models in a context where many of the things that need measurement have inherently global characteristics is not terribly useful. Neither are efforts to develop complementary measures to the GDP even at global scale, arguments for which have lingered in the academic and policy wings since at least the social indicators movement of the 1980s and 1990s and even earlier to the 1960s. The 2013 Organisation for Economic Co-operation and Development (OECD) Framework for Well-being (based on the 2009 Report by the Commission on the Measurement of Economic Performance and Social Progress)^22^ is perhaps the most cogent attempt to gain consensus on a global “dashboard” of measures.^23^ Others have suggested that consideration be given to the (more or less agreed upon) metrics of the non-mandatory Sustainable Development Goals that member states use in their own intermittent voluntary reporting,^24^ notwithstanding the inherent contradictions between goals related to the economy and those emphasizing the environment.

 A commentary on the WHO Council on the Economics of Health For All, which also calls for a different set of metrics “beyond GDP,” maintains that the “stickiness” of GDP and its still politically dominant growth mantra should lead us to amend the metrics of the UN System of National Accounts, on which the GDP is based.^25^ Revisions should ensure that stocks as well as flows are measured, and that aspects of the OECD Framework capturing inequality indicators, environmental impacts, and a broad array of social impacts form part of the summary and disaggregated reporting.

## Déjà vu or De Novo?

 In many ways the rise of the idea of well-being economies is *déjà vu* for activist public health movements that in the recent past mobilized around “healthy cities,” “sustainable cities” or other efforts to shape municipal policies. These initiatives persist, their sectoralism (health in one instance, environment in another) became niche movements, important but limited. The same fate may befall the doughnut and WEALL hubs, although the well-being economy (importantly the two words as inseparable concept) embeds a trenchant critique of the economic status quo. It takes a stand against the destructive excess consumption of some at the impoverishment of others and wasting of life-essential environmental resources. But:

 “*…unlike other critiques of the growth economy that project an image of contraction, parsimony and deprivation, the WE (well-being economy) uses a ‘positive language’ of abundance, wellness and conviviality, with a view to building a forward-looking narrative of opportunities for human creativity, thus inspiring collective action and making governments more amenable to policy change.*”^26^

 With a strong emphasis on living in harmony with nature a well-being economy has global resonance, from the Latin-American *buen vivir*to the South African *ubuntu*, the Swedish *lagom*, and values associated with Buddhism and Confucianism. It is also very consonant with the (critical) public health literature on social determinants of health, and the long-standing community empowerment tenets of many health promotion and social welfare programs and practices. In that sense, the current promotion of well-being economics is something both ancient (and hence renewed) but also new (embedding within it an implicit or occasionally explicit critique of status quo economics, with an emphasis on collective and not just individual well-being).

## But What of the Powerful?

 All the well-being economy initiatives so far described make powerful statements aimed at reforming capitalism into something more humane, equitable, and environmentally sustainable. None of them explicitly question whether the capitalist economy is capable of institutionalizing such reforms, although one of the E4A lead authors, Sandrine Dixson-Declève, in an interview, was more direct: “Are we going against capitalism and the neoliberal model? Yes…” What remains unstated in the well-being economy discourse is how to overcome the opposition of powerful economic actors and elites whose self-interest might allow small, mitigating well-being measures, but challenge anything that threatens to reduce their own wealth and privileges. Simply changing the metrics of our public accounts to incorporate well-being indicators is insufficient as a change strategy. True: a small group of millionaires, perhaps also some billionaires, have signed a statement asking for fairer taxation, on income and wealth, the starting point (alongside corporate regulation and breaking up monopolies) for a well-being economy.^27^ Most signatories, though, are the millionaires, and none of the wealthiest of the billionaires have signed on to this statement.

 There are now UN-level negotiations to create a framework convention on tax that “could deliver the biggest shake-up in history to the global tax system,”^28^ and at a minimum should be vigorously supported by any government making well-being economy claims. So, too, should be well-being government reporting on progress in implementing the well-being economy policy measures listed in Table.

 But what of the capitalism/consumption contradiction that, by its inherent market logic, cannot escape a trajectory of unsustainable consumption? Plotting some escape routes out of capitalism is the biggest and most urgent challenge facing efforts to put well-being economics into substantive practice. Governments here will need the creativity and advocacy of a strong, mobilized civil society working alongside committed policy and political actors willing to confront powerful opponents. Self-described well-being economy countries will need to protect democratic space for such resistance, and oppose the right-ward drift to autocratic and theocratic regimes.

## Ethical issues

 Not applicable.

## Competing interests

 Author declares that he has no competing interests.
